# Supplementary material for: Insights into the Genetic Architecture and Genomic Prediction of Powdery Mildew Resistance in Flax (Linum usitatissimum L.)
Source: Int J Mol Sci. 2022 Apr 29;23(9):4960. doi: 10.3390/ijms23094960 (PMC9104541; doi:10.3390/ijms23094960)
Supplement: Supplementary file 1 [file ijms-23-04960-s001.zip › FigureS2.pdf]

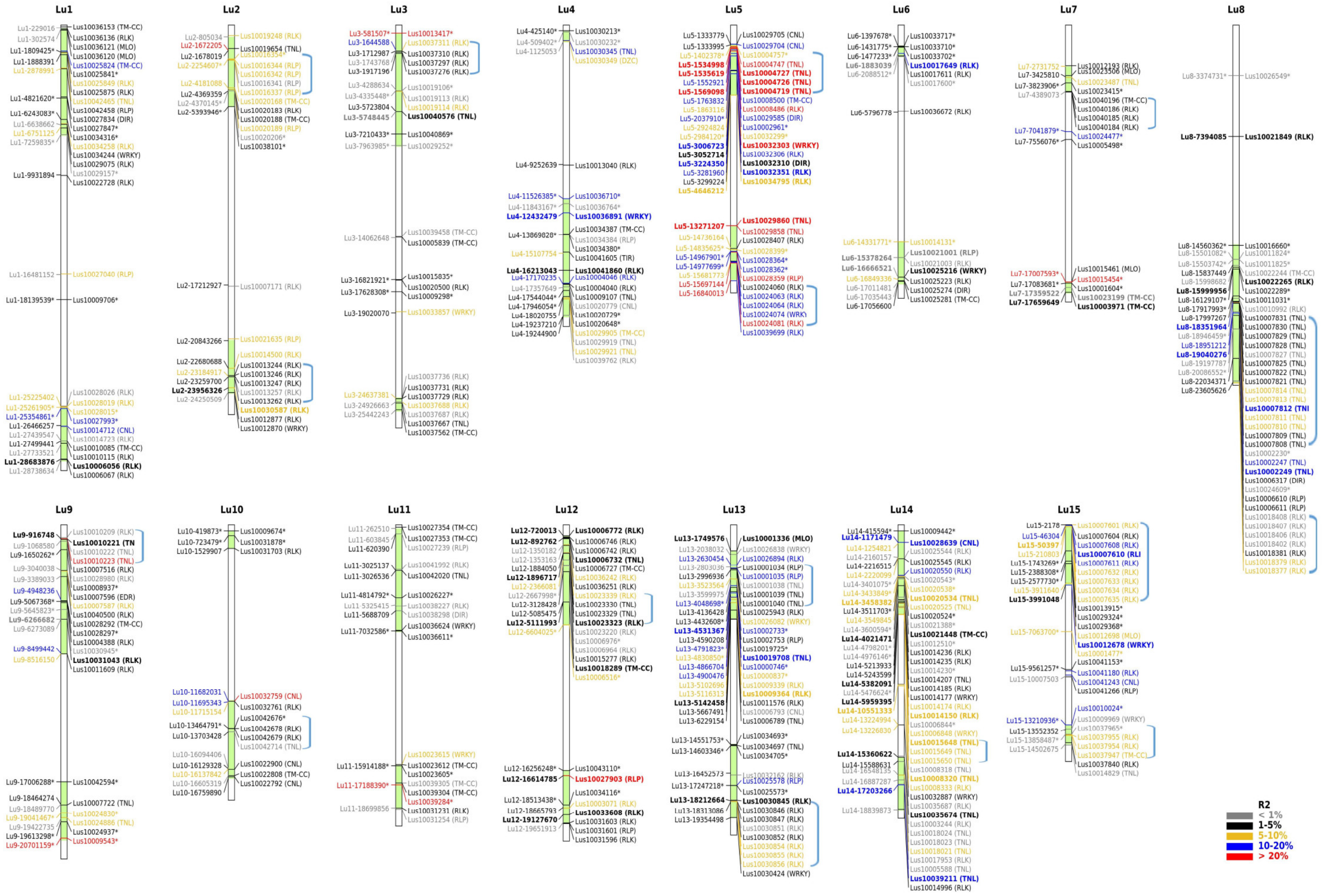

Figure S2. Chromosome distribution of 445 candidate resistance gene analogs (RGA) and 87 non-RGAs (labeled with '*') co-located with 269 quantitative trait nucleotides (QTNs) within 200 Kb genome regions of the QTNs. R^2 represents the proportion of phenotypic variation explained by a QTN or a SNP on a candidate gene. The brackets show candidate gene clusters that contain at least three genes.
