# Supplementary material for: Insights into the Genetic Architecture and Genomic Prediction of Powdery Mildew Resistance in Flax (Linum usitatissimum L.)
Source: Int J Mol Sci. 2022 Apr 29;23(9):4960. doi: 10.3390/ijms23094960 (PMC9104541; doi:10.3390/ijms23094960)
Supplement: Supplementary file 1 [file ijms-23-04960-s001.zip › FigureS3.pdf]

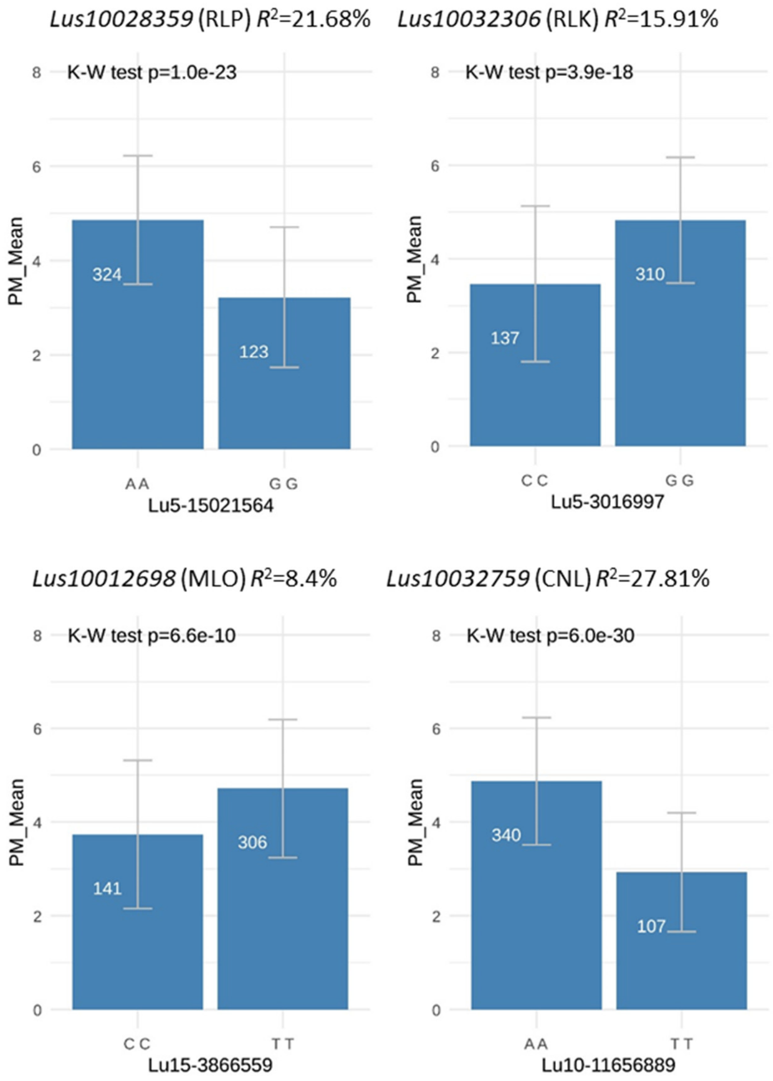

Figure S3. Significant allele effects of SNPs on four candidate resistance gene analogs (RGAs), an examples of candidate RGAs that was supported by at least one SNP with significant SNP allele effects. SNP with chromosome-position is indicated below each graph and gene, R^2 and P value of the Kruskal-Wallis tests are above
