# Supplementary material for: Insights into the Genetic Architecture and Genomic Prediction of Powdery Mildew Resistance in Flax (Linum usitatissimum L.)
Source: Int J Mol Sci. 2022 Apr 29;23(9):4960. doi: 10.3390/ijms23094960 (PMC9104541; doi:10.3390/ijms23094960)
Supplement: Supplementary file 1 [file ijms-23-04960-s001.zip › FigureS4.pdf]

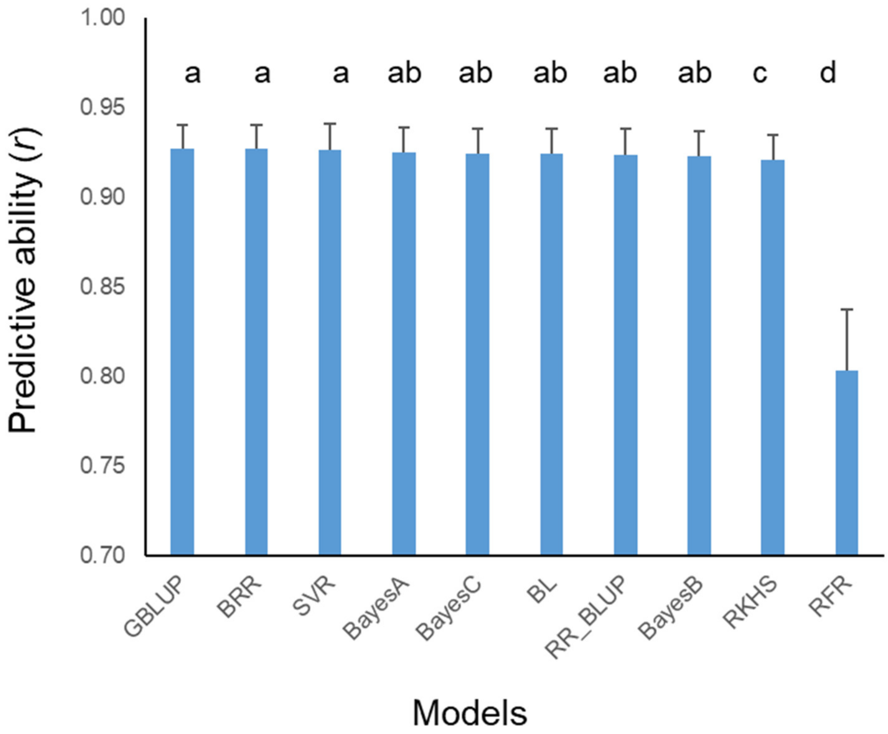

Figure S4. Comparisons of predictive ability among ten genomic selection models using the mean dataset of powdery mildew (PM) ratings over five years (2012-2017) and 349 quantitative trait nucleotides (QTNs). Although the performance of RKHS looks similar with the other eight models except for RFR, the statistical test indicated significant difference from other models. The different letters on the top of bars represent statistical significance at 5% probability level using the Tukey HSD test.
