# Supplementary material for: Insights into the Genetic Architecture and Genomic Prediction of Powdery Mildew Resistance in Flax (Linum usitatissimum L.)
Source: Int J Mol Sci. 2022 Apr 29;23(9):4960. doi: 10.3390/ijms23094960 (PMC9104541; doi:10.3390/ijms23094960)
Supplement: Supplementary file 1 [file ijms-23-04960-s001.zip › FigureS5.pdf]

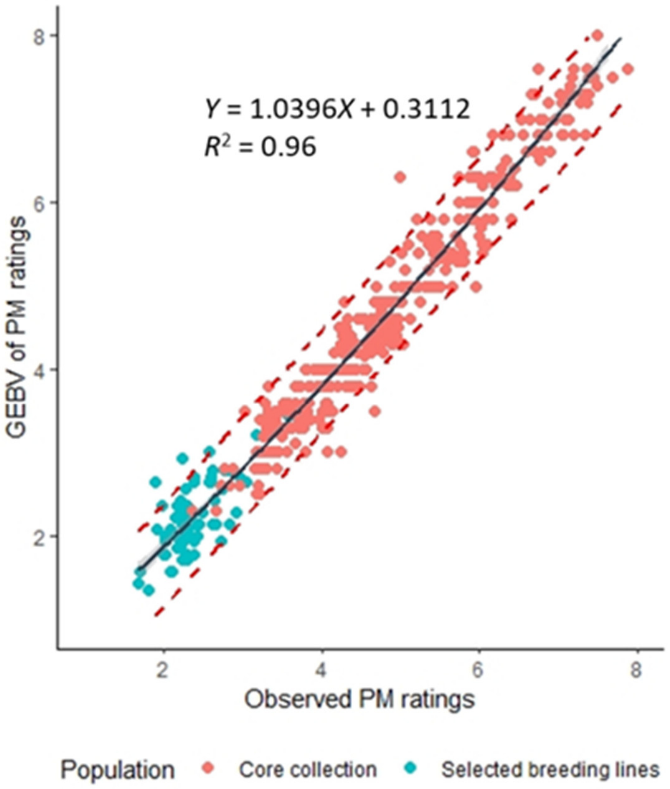

Figure S5. Relationship between the mean powdery mildew (PM) ratings over five years and the genomic estimated breeding value (GEBV) predicted by a GBLUP model constructed using 349 quantitative trait nucleotides (QTNs) and 447 accessions.
