# Supplementary material for: Insights into the Genetic Architecture and Genomic Prediction of Powdery Mildew Resistance in Flax (Linum usitatissimum L.)
Source: Int J Mol Sci. 2022 Apr 29;23(9):4960. doi: 10.3390/ijms23094960 (PMC9104541; doi:10.3390/ijms23094960)
Supplement: Supplementary file 1 [file ijms-23-04960-s001.zip › TableS1.pdf]

Table S1. Powdery mildew (PM) ratings of the 75 selected breeding lines across 8 years (2010-2017).

**Year**	$\bar{x} \pm s$	**CV (%)**
2010	1.70 ± 0.75	44.12
2011	3.24 ± 1.12	34.57
2012	2.59 ± 0.90	34.75
2013	2.41 ± 0.98	40.66
2014	2.27 ± 0.95	41.85
2015	2.39 ± 0.78	32.64
2016	2.24 ± 0.91	40.63
2017	2.01 ± 0.77	38.31
Mean	2.34 ± 1.00	42.74
