# Supplementary material for: Insights into the Genetic Architecture and Genomic Prediction of Powdery Mildew Resistance in Flax (Linum usitatissimum L.)
Source: Int J Mol Sci. 2022 Apr 29;23(9):4960. doi: 10.3390/ijms23094960 (PMC9104541; doi:10.3390/ijms23094960)
Supplement: Supplementary file 1 [file ijms-23-04960-s001.zip › TableS2.pdf]

Table S2. Haplotype blocks identified from 247,160 single nucleotide polymorphisms (SNPs) in the core collection and selected breeding lines.

Chr[1]	No of SNPs	No of Blocks (≥ 2 SNPs)	No of Singletons	Average SNPs per Block	Average Block Size (Kb) -Exclude Singletons	Min – Max Size (Kb)
Lu1	23, 584	4, 503	8, 691	3.31	2.00 ± 4.58	0.003 ± 112.851
Lu2	17, 884	3, 389	6, 408	3.39	1.91 ± 5.85	0.003 ± 172.352
Lu3	19, 564	3, 719	7, 568	2.23	2.09 ± 5.40	0.003 ± 119.824
Lu4	12, 387	2, 383	5, 134	3.04	2.09 ± 4.59	0.003 ± 59.76
Lu5	9, 251	1, 778	5, 205	2.28	1.73 ± 3.71	0.003 ± 60.963
Lu6	17, 377	3, 282	6, 471	3.32	1.81 ± 3.83	0.003 ± 68.92
Lu7	16, 570	3, 242	6, 131	3.22	1.69 ± 3.87	0.003 ± 90.713
Lu8	14, 151	2, 774	5, 719	3.04	2.46 ± 4.46	0.003 ± 157.511
Lu9	17, 417	3, 349	6, 149	3.36	1.91 ± 4.81	0.003 ± 105.901
Lu10	13, 145	2, 463	5, 154	3.24	2.28 ± 4.59	0.003 ± 73.838
Lu11	14, 284	2, 748	5, 433	3.22	1.90 ± 4.33	0.003 ± 114.884
Lu12	19, 046	3, 743	6, 669	3.31	1.79 ± 4.00	0.003 ± 64.149
Lu13	21, 242	4, 064	8, 038	3.25	1.81 ± 4.48	0.003 ± 106.751
Lu14	17, 254	3, 341	6, 316	3.27	1.73 ± 4.08	0.003 ± 77.431
Lu15	14, 004	2, 786	5, 088	3.20	1.77 ± 5.12	0.003 ± 99.929
**Total**	**247, 160**	**47, 564**	**94, 174**	**3.22**	**1.93 ± 4.72**	**0.003 ± 172.352**

[1] Chr: chromosome
