# Supplementary material for: Insights into the Genetic Architecture and Genomic Prediction of Powdery Mildew Resistance in Flax (Linum usitatissimum L.)
Source: Int J Mol Sci. 2022 Apr 29;23(9):4960. doi: 10.3390/ijms23094960 (PMC9104541; doi:10.3390/ijms23094960)
Supplement: Supplementary file 1 [file ijms-23-04960-s001.zip › TableS4.pdf]

Table S4. Quantitative trait nucleotides (QTNs) associated with powdery mildew (PM) ratings identified using two single locus and seven multi-locus models from seven phenotype data sets. No QTNs were detected by MLM.

Statistical Model	No. of QTL/QTNs Identified	Average R^2 (%)	Range of R^2 (%)
GLM	9	11.34	0.65 – 27.62
FASTmrEMMA	68	4.24	0.02 – 21.28
FASTmrMLM	105	5.25	0.02 – 29.67
ISIS EM-BLASSO	83	3.66	0.02 – 17.28
mrMLM	72	4.74	0.02 – 26.93
pKWmEB	90	3.19	0.01 – 26.93
pLARmEB	108	5.79	0.02 – 30.20
FarmCPU	15	9.07	0.04 – 27.62
**Total unique**	**349**	**4.53**	**0.01 – 30.20**
