# Supplementary material for: Insights into the Genetic Architecture and Genomic Prediction of Powdery Mildew Resistance in Flax (Linum usitatissimum L.)
Source: Int J Mol Sci. 2022 Apr 29;23(9):4960. doi: 10.3390/ijms23094960 (PMC9104541; doi:10.3390/ijms23094960)
Supplement: Supplementary file 1 [file ijms-23-04960-s001.zip › TableS5.pdf]

Table S5. The number of common quantitative trait nucleotides (QTNs) with powdery mildew (PM) ratings identified by any two statistical models.

**Methods**	**GLM**	**FASTmr MLM**	**FASTmr EMMA**	**ISIS EM-BLASSO**	**mrMLM**	**pKWmEB**	**pLARmEB**
FASTmrMLM	2						
FASTmrEMMA	2	19					
ISIS EM-BLASSO	4	20	13				
mrMLM	2	22	16	17			
pKWmEB	4	19	15	29	18		
pLARmEB	2	39	19	14	20		
FarmCPU	3	3	0	3	2	20	1
