# Supplementary material for: Insights into the Genetic Architecture and Genomic Prediction of Powdery Mildew Resistance in Flax (Linum usitatissimum L.)
Source: Int J Mol Sci. 2022 Apr 29;23(9):4960. doi: 10.3390/ijms23094960 (PMC9104541; doi:10.3390/ijms23094960)
Supplement: Supplementary file 1 [file ijms-23-04960-s001.zip › TableS6.pdf]

Table S6. Quantitative trait nucleotides (QTNs) identified from various phenotypic datasets.

**Phenotype Dataset**	**No. of QTNs Identified**	**Average R^2 (%)**	**Range of R^2 (%)**
PM-2012	45	4.61	0.03 – 20.89
PM-2013	73	6.57	0.02 – 30.20
PM-2014	84	4.17	0.02 – 21.28
PM-2015	64	4.34	0.02 – 29.67
PM-2016	43	2.92	0.01 - 15.67
PM-Mean	63	4.76	0.01 – 26.93
