# Supplementary material for: Insights into the Genetic Architecture and Genomic Prediction of Powdery Mildew Resistance in Flax (Linum usitatissimum L.)
Source: Int J Mol Sci. 2022 Apr 29;23(9):4960. doi: 10.3390/ijms23094960 (PMC9104541; doi:10.3390/ijms23094960)
Supplement: Supplementary file 1 [file ijms-23-04960-s001.zip › TableS7.pdf]

Table S7. Common quantitative trait nucleotides (QTNs) which were identified from two powdery mildew (PM) datasets.

Phenotype Dataset	PM-2012	PM-2013	PM-2014	PM-2015	PM-2016
PM-2013	0				
PM-2014	0	1			
PM-2015	0	0	1		
PM-2016	0	0	1	1	
PM-Mean	3	2	9	8	1
