# Supplementary figures and images for: Insights into the Genetic Architecture and Genomic Prediction of Powdery Mildew Resistance in Flax (Linum usitatissimum L.)
Source: Int J Mol Sci. 2022 Apr 29;23(9):4960. doi: 10.3390/ijms23094960 (PMC9104541; doi:10.3390/ijms23094960)

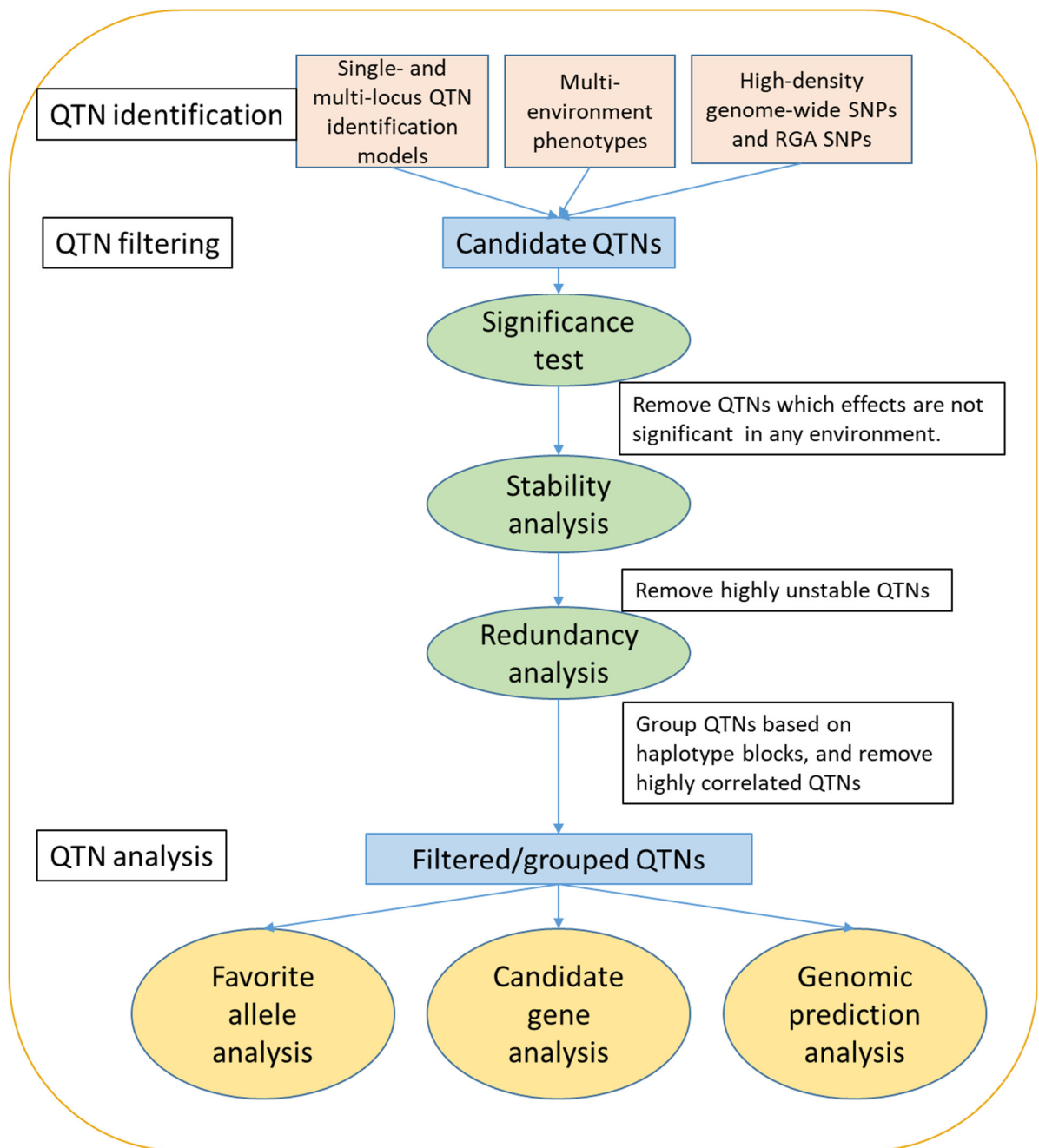

**Figure S6.** Pipeline for quantitative trait nucleotide (QTN) identification and analysis

Supplement: Supplementary file 1 [file ijms-23-04960-s001.zip › FigureS6.pdf]
